# Overexpression of ALS-Associated p.M337V Human TDP-43 in Mice Worsens Disease Features Compared to Wild-type Human TDP-43 Mice

**DOI:** 10.1007/s12035-013-8427-5

**Published:** 2013-03-10

**Authors:** Jonathan Janssens, Hans Wils, Gernot Kleinberger, Geert Joris, Ivy Cuijt, Chantal Ceuterick-de Groote, Christine Van Broeckhoven, Samir Kumar-Singh

**Affiliations:** 1 Neurodegenerative Brain Diseases Group, Department of Molecular Genetics, VIB, Universiteitsplein 1, 2610 Antwerp, Belgium; 2 Present Address: Molecular and Cellular Neuropathology Group, Laboratory of Cell Biology and Histology, Translational Neuroscience Department, Faculty of Medicine, University of Antwerp-CGB, Groenenborgerlaan 171, 2020, Antwerp, Belgium; 3 Laboratory of Neurogenetics, University of Antwerp, Universiteitsplein 1, 2610 Antwerp, Belgium; 4 Laboratory of Ultrastructural Neuropathology, Institute Born-Bunge, University of Antwerp, 2610 Antwerp, Belgium

**Keywords:** Amyotrophic lateral sclerosis, Frontotemporal lobar degeneration, *TARDBP*, Transgenic mice, Ubiquitin

## Abstract

Mutations in TAR DNA-binding protein 43 (TDP-43) are associated with familial forms of amyotrophic lateral sclerosis (ALS), while wild-type TDP-43 is a pathological hallmark of patients with sporadic ALS and frontotemporal lobar degeneration (FTLD). Various in vitro and in vivo studies have also demonstrated toxicity of both mutant and wild-type TDP-43 to neuronal cells. To study the potential additional toxicity incurred by mutant TDP-43 in vivo, we generated mutant human TDP-43 (p.M337V) transgenic mouse lines driven by the Thy-1.2 promoter (Mt-TAR) and compared them in the same experimental setting to the disease phenotype observed in wild-type TDP-43 transgenic lines (Wt-TAR) expressing comparable TDP-43 levels. Overexpression of mutant TDP-43 leads to a worsened dose-dependent disease phenotype in terms of motor dysfunction, neurodegeneration, gliosis, and development of ubiquitin and phosphorylated TDP-43 pathology. Furthermore, we show that cellular aggregate formation or accumulation of TDP-43 C-terminal fragments (CTFs) are not primarily responsible for development of the observed disease phenotype in both mutant and wild-type TDP-43 mice.

**Electronic supplementary material:**

The online version of this article (doi:10.1007/s12035-013-8427-5) contains supplementary material, which is available to authorized users.

## Introduction

TAR DNA-binding protein 43 (TDP-43) is the major pathological inclusion protein involved in the pathogenesis of amyotrophic lateral sclerosis (ALS) and frontotemporal lobar degeneration (FTLD) linked to TDP-43 pathology (FTLD-TDP), providing a molecular link that put these disorders in a common ALS-FTLD disease spectrum [1–3]. ALS is the most common adult-onset form of motor neuron disease and is characterized by a degeneration of cortical motor neurons and anterior horn cells of the spinal cord. FTLD-TDP, on the other hand, is the most common pathological subtype of FTLD [1, 2], the latter only being preceded in prevalence by Alzheimer’s disease [3, 4]. Numerous causal missense mutations in the TDP-43 gene (*TARDBP*) have been reported in patients with ALS (AD&FTLD Mutation Database; http://www.molgen.ua.ac.be/Admutations/) [5–8]. TDP-43 is a highly conserved, ribonuclear protein with two RNA recognition motifs (RRM1 and RRM2) and a glycine-rich C-terminal domain that mediates protein–protein interactions [9]. Nearly all of the TDP-43 mutations are localized within the glycine-rich domain of TDP-43 indicating a high importance for this domain in disease development [7]. Several functions of TDP-43 have been described such as a role in transcription, RNA splicing, microRNA biogenesis and development [10]. In addition, comprehensive studies aimed at identifying RNA-binding targets for TDP-43 found a multitude of target RNAs including transcripts of genes involved in RNA metabolism, synaptic function, and central nervous system (CNS) development [11]. However, it remains unclear which of these functions, if any, are hampered by aberrant TDP-43 and trigger disease development [12].

TDP-43 pathology in ALS and FTLD-TDP patients is characterized by abnormal protein processing including ubiquitination, phosphorylation, and proteolysis generating C-terminal fragments (CTFs) [1, 2, 13]. The abnormal TDP-43 species are sequestered in both cytoplasmic and nuclear protein aggregates, which are invariably associated with substantial depletion of nuclear full-length TDP-43, a phenomenon called “nuclear clearing” [1, 2]. Recent studies have indicated that the TDP-43 C-terminus contains a “prion-like” domain that makes full-length TDP-43 or its CTFs aggregation prone [14, 15]. Although toxicity of CTFs and aggregate formation has been shown before [16, 17], it is currently unknown whether these TDP-43 species are mechanistically linked to neurodegeneration or constitute a secondary event in the disease cascade. Furthermore, additional toxicity incurred by *TARDBP* mutations compared to wild-type TDP-43 in the development of ALS-FTLD diseases also needs further clarification. Several animal models overexpressing either mutant or wild-type TDP-43 have been reported (Table 2) to develop a highly similar ALS-FTLD-like phenotype [18–28]. To investigate the potential additional toxicity incurred by mutant TDP-43 on the integrity of neuronal cells, we developed various germline transgenic mouse lines overexpressing human TDP-43 containing the methionine-to-valine substitution (p.M337V) occurring in familial ALS patients [6]. Transgene expression was driven by the murine Thy-1.2 promoter and transgenic lines were chosen to have comparable TDP-43 levels as previously reported by us for wild-type TDP-43 mice [24]. Compared to wild-type TDP-43 mice, overexpression of mutant TDP-43 leads to a worsened dose-dependent ALS-FTLD-like phenotype in terms of motor dysfunction and lethality, neurodegeneration, and gliosis as well as development of phosphorylated TDP-43 cytoplasmic granules. We also show that accumulation of CTFs, together with formation of aggregates do not seem to be associated with the development of the observed ALS-FTLD-like phenotype.

## Material and Methods

### Generation of Mutant (p.M337V) Human TDP-43 Overexpression Mice

Mice overexpressing mutant human TDP-43 were developed using *TARDBP* cDNA amplified from a human cDNA library and cloned into a Thy-1.2 expression vector (mTUB, QPS JSW Life Sciences GmbH; www.jsw.lifesciences.com) containing a modified murine Thy-1.2 promoter [24]. The p.M337V missense mutation was introduced by standard Polymerase Chain Reaction (PCR) mutagenesis (QuikChange®, Stratagene) and the sequence was verified by Sanger sequencing. The expression vector was subsequently microinjected into pronuclear oocytes of Bl6/SJL mice by the Yale Transgenic Mouse Service Facility (New Haven, CT, USA). Offspring were genotyped and 11 transgenic pups carried the transgene. Founder mice were backcrossed to C57Bl6/J up to five generations to establish stable transgenic mouse lines. Furthermore, hemizygous crossbreedings were performed to obtain homozygous mutant TDP-43 overexpressing mice. Zygosity was determined by Multiplex Amplicon Quantification (MAQ, Multiplicom) assays and PCR (PCR primers available on request). Homozygous expression levels were confirmed by semiquantitative real-time PCR (qRT-PCR) on brain tissue of 2-week-old mice. All animal experiments were approved by the University of Antwerp Ethics Committee and conducted according to the guidelines of the Federation of European Laboratory Animal Science Associations (FELASA) and the EU Directive 2010/63/EU for animal experiments.

### Gait Analysis

Motor coordination and balance were assessed by accelerating rotarod and footprint analysis, as described previously [24]. Briefly, accelerating rotarod (Rota-Rod Treadmill; Med Associates Inc.) was performed for three subsequent runs and repeated every month. Gradual acceleration of the rotating rod ranged from 2.5 to 25 rpm and 3.5 to 35 rpm, with a maximum observation time of 5 min. Time spent on the rod was automatically recorded by interrupting a photobeam on the rotarod floor.

Limb motor function was assessed by footprint analysis where fore- and hindpaws were dipped in red and black nontoxic paints, respectively. Subsequently, mice were allowed to walk down a narrow runway (50 cm long, 7 cm wide, and flanked by 10 cm high walls) with a white paper covering the floor. Paw position and stride width and length as well as the paw progression angle (PPA) were measured and compared to non-transgenic (Ntg) littermates.

### Tissue Harvesting and Processing

Brains were harvested, weighed, and cut midsagittally according to standard protocol [29]. Right hemispheres were snap frozen in liquid nitrogen and stored at −80 °C for subsequent mRNA and protein analysis. Left hemispheres were fixed in 2 % paraformaldehyde (PFA) for 18–20 h and prepared for paraffin embedding. Spinal cords were processed similarly as described for brain. Cerebral and lumbar parts of the spinal cord were cut and fixed in 4 % glutaraldehyde for 4 h for electron microscopy analysis.

### Semiquantitative Real-time PCR

To determine the expression levels of human and mouse TDP-43, total RNA was isolated from murine brain using the RiboPure™ kit followed by a DNase treatment (TURBO DNase Kit; both Ambion). First-strand cDNA was synthesized using the SuperScript® III First-Strand Synthesis System (Life Technologies) using random hexamer primers. Expression was measured using TaqMan® MGB assays (human and mouse TDP-43) designed with File Builder software (Applied Biosystems) or SYBR Green technology (cathepsin D and caspase-3) on an ABI ViiA™ 7 Real-Time PCR System (Applied Biosystems). Quantification of transcript levels was achieved with glyceraldehyde 3-phosphate dehydrogenase (GAPDH) and β-actin as housekeeping controls (primer and probe sequences available on request), as described previously [30]. Each sample was measured in duplicate and at least two independent experiments were performed.

### Immunoblotting

Proteins from brain tissue were prepared as low-salt and 2 % sodium dodecyl sulfate (SDS) fractions using cell lysis buffer (CLB; 10 mM HEPES, 10 mM NaCl, 1 mM KH_2_PO_4_, 5 mM NaHCO_3_, 5 mM ethylenediaminetetraacetic acid (EDTA), 1 mM CaCl_2_, 0.5 mM MgCl_2_, 10× volume/weight (V/wt)) followed by radioimmunoprecipitation assay (RIPA) buffer supplemented with 2 % SDS (5× V/wt), as described previously [24]. All reported buffers were supplemented with protease (complete protease inhibitor cocktail, Roche) and phosphatase (PhosphoSTOP, Roche) inhibitors.

Protein content of low-salt fractions was determined with a bicinchoninic acid colorimetric assay (Perbio Science N.V.). Equal protein amounts were loaded and separated on 10 % Nupage® Bis–Tris gels (Life Technologies) and electroblotted onto a polyvinylidene difluoride membrane (Hybond P, PVDF; Amersham Biosciences). After blotting, membranes were blocked in 5 % skimmed milk in phosphate-buffered saline (PBS) containing 0.1 % Tween® 20 (Merck). Membranes were probed with a range of primary antibodies listed in Table S1. Immunodetection was achieved using horseradish peroxidase (HRP)-conjugated secondary antibodies and an ECL Plus™ chemiluminescent detection system (GE Healthcare). Bands were quantified on a Kodak Imaging Station 440 (Eastman Kodak) and quantitative data were normalized to GAPDH for low-salt fractions (Meridian Life Science) or lamin A/C for 2 % SDS fractions (clone N-18, Santa Cruz Biotechnology), as described earlier [24].

### Histology and Immunohistochemistry

For each specimen, 4-μm thick sections were cut using an automated HM355 Microm rotary microtome (Microm International). Classical histochemistry was performed according to standard protocols [31]. For immunohistochemistry (IHC), sections were deparaffinized, rehydrated, pretreated with citrate buffer to enhance immunoreactivity, and developed using 3,3′-diaminobenzidine (DAB). Primary antibodies used for IHC are listed in Table S1. Sections were counterstained with hematoxylin. Images were taken on an Axioskop 50 light microscope (Zeiss) equipped with a CCD UC30 camera (Olympus Inc.). Double-labeling immunofluorescence was performed on brain sections using a TDP-43 non-species-specific (Proteintech Group) or a phosphorylated TDP-43 (Cosmo Bio; pS409/pS410 or pS403/404) specific antibody together with a TIA-1 (Santa Cruz Biotechnology) or monoclonal ubiquitin (Life Technologies) antibody, and visualized with secondary antibodies conjugated to Alexa Fluor® 488 or Alexa Fluor® 594 (Life Technologies). 4′,6-Diamidino-2-phenylindole (DAPI; Bio-Rad) was used as a nuclear counterstain. Images were taken on a LSM700 confocal microscope (Zeiss). For quantification of gliosis, 40× images were taken in the cortical layer V of the brain and the anterior horn of the spinal cord from Ntg (*n* = 3) and Mt-TAR6/6 (*n* = 3) mice. Immunoreactive astrocytes and glial cells were counted manually and normalized to the area.

### Electron Microscopy

Ultrastructural microscopy was performed on cerebral and lumbar regions of the spinal cord from transgenic and Ntg control animals. Tissue was fixed in 4 % buffered glutaraldehyde for 4 h, and 2 % osmium tetraoxide before embedding and processing according to standard protocols [31, 32]. Sections were contrasted with routine uranyl acetate and lead citrate and analyzed by a Philips CM10 electron microscope equipped with a goniometric coordinator as reported previously [31].

### Statistical Analysis

All experiments were performed in duplicate and repeated at least two times with results reported as mean ± standard deviation (SD). *P* values for description of statistical significance of differences were calculated by two-tailed Student’s *t* tests if not mentioned otherwise. *P* < 0.05 was considered significant.

## Results

### Mutant (p.M337V) TDP-43 Expression is Comparable to Transgene Levels in Wild-Type TDP-43 Overexpression Mice

**Fig. 1:**
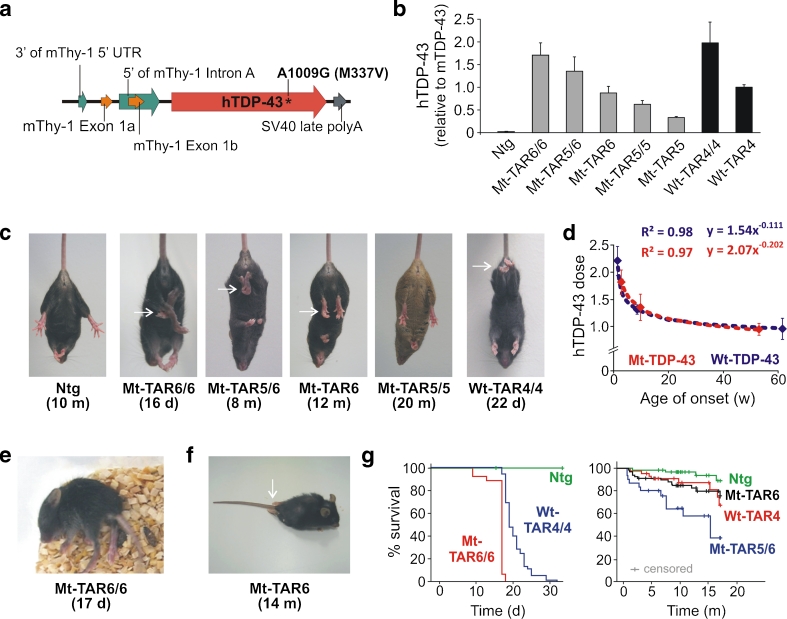
Overexpression of mutant human TDP-43 leads to a dose-dependent motor phenotype. **a** Modified Thy-1.2 expression vector used to generate mutant (p.M337V) human TDP-43 (hTDP-43) transgenic mice. **b** *TARDBP* mRNA expression levels measured in brain by qRT-PCR for the different mutant TDP-43 (Mt-TAR) mouse lines. Mt-TAR6/6 expression levels were comparable to those measured in wild-type TDP-43 mice (Wt-TAR4/4 and Wt-TAR4) as described earlier. **c** Overexpression of mutant TDP-43 induced an abnormal hindlimb reflex (*arrows*) at variable ages dependent on the TDP-43 dose that was absent in non-transgenic (Ntg) mice and Mt-TAR5/5 mice expressing a low TDP-43 dose. **d** TDP-43 dose for both mutant (Mt-TAR6/6, *n* = 8; Mt-TAR5/6, *n* = 7; Mt-TAR6, *n* = 12) and wild-type (Wt-TAR4/4, *n* = 6; Wt-TAR4, *n* = 8; Wt-TAR6/6, *n* = 4) TDP-43 mice showed a log–log correlation with the age of onset or the age where the abnormal hindlimb reflex was first noticed (*R* ^2^ = 0.97). **e** Typical example of an end-stage paralysis in Mt-TAR6/6 mice (Movie S1). **f** About 5 % of end-stage hemizygous Mt-TAR6 mice developed severe paralysis of the hindlimbs and acquired a swimming gait. **g** Kaplan–Meier analysis for different mutant and wild-type TDP-43 mice revealing a reduced lifespan for transgenic mice compared to Ntg littermates. Homozygous mutant TDP-43 mice show higher mortality compared to wild-type TDP-43 mice, which is reflected by an average survival of 17 days for Mt-TAR6/6 compared to 24 days for Wt-TAR4/4 mice (Mt-TAR6/6, *n* = 29; Wt-TAR4/4, *n* = 49). Double hemizygous Mt-TAR5/6 mice have an average survival of 9.3 months compared to 16.4 and 16.6 months for Mt-TAR6 and Wt-TAR4 mice, respectively (Ntg, *n* = 69; Mt-TAR6, *n* = 72; Wt-TAR4, *n* = 46; Mt-TAR5/6, *n* = 31). [24]

We previously demonstrated that overexpression of wild-type human TDP-43 under control of a neuronal murine Thy-1.2 promoter leads to a dose-dependent ALS-FTLD-like motor phenotype in two independent mouse lines (TAR4/4 and TAR6/6; hereafter called Wt-TAR4/4 and Wt-TAR6/6) [24]. To compare potential additional toxicity of mutant TDP-43 with wild-type TDP-43, we generated multiple mouse lines for mutant (p.M337V) TDP-43 using the same promoter (Fig. 1a). The choice of the promoter for both mutant and wild-type lines was driven by two important requirements. First, because TDP-43 aggregates are majorly present in neurons, the use of a neuronal Thy-1.2 promoter was preferred for these experiments as it drives expression preferentially in neurons. Second, the Thy-1.2 promoter becomes active approximately 1 week after birth [33] that reduces the risk of interfering with essential functions of TDP-43 during embryonic development [5].

Eleven transgenic founders for mutant p.M337V TDP-43 mice were generated that showed germline transmission of mutant TDP-43. To match expression levels with those of the previously reported hemi- and homozygous wild-type TDP-43 mice [24], different homozygous and double hemizygous mutant TDP-43 mice were generated. Two lines (Mt-TAR5 and Mt-TAR6) were selected that showed 0.34- and 0.88-fold mutant TDP-43 expression compared to endogenous transcript levels analyzed by qRT-PCR on brain tissue. In addition, homozygous Mt-TAR5/5, double hemizygous Mt-TAR5/6, and homozygous Mt-TAR6/6 were generated that showed expected expression levels (Fig. 1b, Fig. S2a). Transgene doses measured in Wt-TAR4 and Wt-TAR4/4 mice were highly comparable to Mt-TAR6 and Mt-TAR6/6 mice, respectively, and TDP-43 levels in Wt-TAR6/6 mice were similar to Mt-TAR5/6 mice (Table 1). The equivalent expression levels between both mouse models allowed us to make direct comparisons concerning pathogenicity of wild-type versus mutant TDP-43 overexpression with respect to the disease phenotype and TDP-43 pathology.

**Table 1:** Phenotypic comparisons between mutant and wild-type TDP-43 mice

	Mt-TAR6/6	Wt-TAR4/4	Mt-TAR5/6	Wt-TAR6/6	Mt-TAR6	Wt-TAR4
Expression (hTDP-43/mTDP-43)	1.7×	2.0×	1.4×	1.2×	0.9×	1.0×
Age of onset (clasping)	11 days	14 days	Variable	60 days	± 336 days	± 392 days
Paralysis	Progressive (fast)	Progressive (fast)	Progressive	Progressive	Progressive (slow)	Progressive (slow)
Age of death	17–18 days	25–26 days	Variable	Variable	≈ 492 days	≈ 498 days
Cytoplasmic ubiquitin	Inclusions (++)^a^	Inclusions (++)	Inclusions (+++)^a^	Inclusions (+)	Diffuse	Diffuse
Cytoplasmic TDP-43	++	+	+	±	−	−
Phosphorylated TDP-43 (cytoplasm)	++	+	+	−	−	−
Increased gliosis	++++	+++	++	++	+	+
C-terminal fragments	↑25-kDa, ↓35-kDa	↑25-kDa, ↓35-kDa	Present	Present	Present	−
Neurodegeneration	Cx, Sp. cord, CA1-4	Cx, Sp. cord, CA3-4	Cx, Sp. cord, CA3-4	Cx, Sp. cord	±	±

^a^Not present in all mice

### Dose-dependent Motor Phenotype in Mutant TDP-43 Mice is Worsened Compared to Wild-Type TDP-43 Mice

Mutant TDP-43 mice were morphologically indistinguishable from their Ntg littermates at birth. Starting at an age of ≈12 days, however, the highest expressing Mt-TAR6/6 mice developed an abnormal hindlimb reflex when suspended by their tails, whereas Ntg littermates extended their hindlimbs (Fig. 1c). This atypical clasping reflex was also reported in wild-type TDP-43 mice [24] and is considered as one of the earliest symptoms of loss of motor control in different ALS mouse models [34]. The severity of the disease phenotype advanced rapidly for Mt-TAR6/6 mice exhibiting a closed body posture, muscle twitches, and reduced mobility at ≈15 days, which was followed by complete paralysis and death within the next 1–2 days (Fig. 1e and g; Movie S1). In addition, Mt-TAR6/6 mice showed severe postnatal growth retardation, including a significant reduction in body weight (54 %; *P* < 0.001; Fig. S1a) and brain weight (22 %; *P* < 0.001; Fig. S1b) compared to Ntg littermates. Interestingly, the motor phenotype in Mt-TAR6/6 mice is highly comparable to that observed in Wt-TAR4/4 mice. Despite nearly identical transgene expression levels, Wt-TAR4/4 mice consistently showed a mildly delayed age of onset for clasping (≈14 days), paralysis (starting at 22 days), and death (25–26 days) [24] (Table 1 and Fig. 1g).

A similar disease phenotype was also present in the second highest expressing double-hemizygous Mt-TAR5/6 mice. The average age of onset as well as disease severity and progression, however, were highly variable. This was demonstrated by several Mt-TAR5/6 mice dying between 1 and 3 months of age, whereas other mice could age up to 17 months. Nevertheless, even in these so-called escapees, abnormal hindlimb reflex (Fig. 1c) and mild paralysis of the hindlimbs with reduced mobility were present. To confirm these observations, we performed motor coordination and gait analyses for Mt-TAR5/6 mice. On the accelerated rotarod test, a significant impairment in motor coordination and balance of Mt-TAR5/6 mice was observed at 9 months of age (*n* = 7; *P* = 0.037; Fig. S3b) that worsened with aging, and at an age of 15 months, mice showed a 36 % decrease in running time compared to Ntg littermates (*n* = 7; *P* = 0.003; data not shown). A footprint analysis for gait also showed an increased paw progression angle (PPA) of the hindlimbs in contrast to Ntg littermates (Fig. S3a; *P* = 0.003). No significant differences were found in stride length, although a decreasing trend was observed in Mt-TAR5/6 mice (data not shown).

The third highest expressing hemizygous Mt-TAR6 mice lived up to 24 months, although they acquired an abnormal hindlimb reflex at 12 months (Fig. 1c and g). Rotarod data showed impairment in motor function beginning at 13 months of age (*n* = 14; *P* = 0.002; Fig. S3b). These changes were also progressive as the same colony of mice showed a maximum decrease of 36 % in running time at an age of 16 months compared to Ntg littermates (*n* = 14; *P* = 0.004). Despite having an equivalent TDP-43 expression, the age of onset for gait abnormalities was accelerated in Mt-TAR6 mice compared to Wt-TAR4 mice (Fig. S3b). Not surprisingly, Mt-TAR6 mice also showed phenotypic variability with a minority of mice (~5 % of the entire colony), developing complete paralysis of the hindlimbs where animals were unable to hold their body off the ground and use their forelimbs to drag themselves forward (Fig. 1f, Movie S2). In this subset of mice, hindlimb paralysis also ranged from 3 to 14 months, indicating variability in both age of onset and disease progression in these lines. In contrast, no clinical phenotype has been observed for low-expressing Mt-TAR5 and Mt-TAR5/5 mice analyzed up to 18–22 months of age. Furthermore, no differences in motor performance on rotarod were observed for Mt-TAR5/5 mice up to an age of 20 months. Taking average age of onsets for the abnormal hindlimb reflex, the correlation of mutant TDP-43 levels in Mt-TAR6/6, Mt-TAR5/6 and Mt-TAR6 mice is best illustrated by a log–log regression model (correlation coefficient *R* ^2^ = 0.97; Fig. 1d, red curve), which is highly similar to the correlation of wild-type TDP-43 levels measured in Wt-TAR4/4, Wt-TAR4, and Wt-TAR6/6 mice (correlation coefficient *R* ^2^ = 0.98; Fig. 1d, blue curve).

Interestingly, a high degree of variability in age of death was also observed in our second and third highest expressing Wt-TAR6/6 and Wt-TAR4 mice [24] (Table 1), with Wt-TAR6/6 dying between 1 and 19 months and some Wt-TAR4 mice living more than 26 months. While some degree of variability is routinely observed in even inbred mouse strains [35], this high degree of variability in phenotype at lower TDP-43 doses suggests involvement of genetic or epigenetic modifying factors influencing levels of TDP-43. To test this hypothesis, we measured TDP-43 levels in the second highest expressing Mt-TAR5/6 mice that had an early or a later age of onset. We demonstrated small but significantly decreased levels of TDP-43 in mice having a later age of onset (*P* = 0.032; Fig. S2c).

Our data also suggested that although mutant TDP-43 mice displayed a comparable disease phenotype compared to Wt-TDP-43 mice, overexpression of mutant TDP-43 appeared to have an accelerating effect on the age of onset and disease progression. We, therefore, studied whether mutant TDP-43 overexpression could lower the dose of endogenous TDP-43 by autoregulatory mechanisms as suggested recently [36]. Consistent with several studies [18, 25–28], both mutant and wild-type TDP-43 overexpression induced a transgene dose-dependent decrease in endogenous TDP-43 transcripts. The decrease noted in Mt-TAR6/6 and Mt-TAR5/6 lines was 24 % (*P* = 0.005) and 18 % (*P* = 0.055), respectively, compared to Ntg littermates (Fig. S2b). This reduction in endogenous TDP-43 expression was not more than the observed decrease of 26 % (*P* = 0.004) in similarly expressing Wt-TAR4/4 mice, suggesting that pathogenic mutant TDP-43 does not act by decreasing endogenous TDP-43 levels.

### Comparable Selective Vulnerability of Neuronal Subpopulations and Dose-dependent Gliosis in Mutant and Wild-Type TDP-43 Mice

Although the Thy-1.2 promoter drives expression in virtually all neuronal cells of the brain and spinal cord, selective vulnerability was observed for specific neuronal subpopulations including cortical layer V motor neurons, spinal anterior horn motor neurons, CA regions of the hippocampus, and thalamic neurons. This selective vulnerability was highly reminiscent of wild-type TDP-43 mice in which TDP-43 expression was driven by the same promoter [24]. In regions where neuronal loss was easily quantifiable, e.g., the CA regions of the hippocampus, more drastic pathology was observed for mutant TDP-43 mice. For instance, the highest expressing Mt-TAR6/6 mice showed severe neuronal loss in all CA regions of the hippocampus with complete obliteration of CA3 and CA4 fields, while CA1 and CA2 fields were reduced to a single layer of neurons. In contrast, equally expressing Wt-TAR4/4 mice showed complete obliteration of only the CA4 field, whereas CA1 and CA2 fields were relatively unaffected (Fig. S4a).

**Fig. 2:**
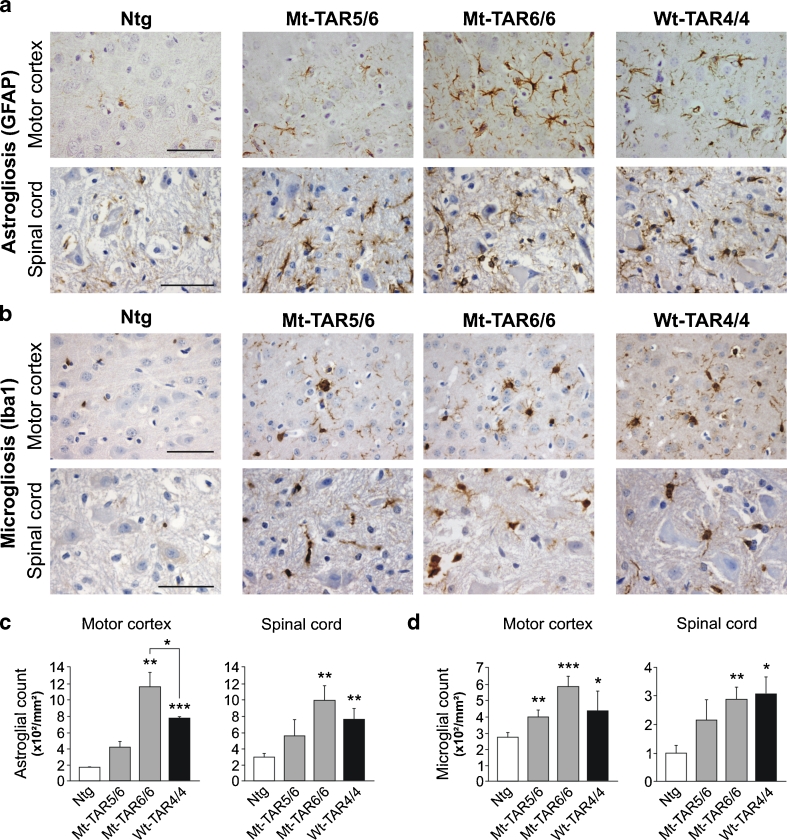
Dose-dependent gliosis in mutant TDP-43 mice. **a** GFAP immunohistochemistry of cortical layer V and spinal cord of Ntg, Mt-TAR6/6, Mt-TAR5/6, and Wt-TAR4/4 mice showing TDP-43 dose-dependent astrogliosis. **b** Similarly, Iba1 immunoreactivity showed a dose-dependent microgliosis in Mt-TAR6/6, Mt-TAR5/6, and Wt-TAR4/4 mice compared to Ntg littermates. **c** Highly increased number of astrocytes in brain and spinal cord of Mt-TAR6/6 and Wt-TAR4/4 mice compared to Ntg littermates. **d** Significantly increased number of activated microglia in brain and spinal cord of Mt-TAR6/6 and WT-TAR4/4 mice compared to Ntg littermates. Overall, mutant TDP-43 induced a more pronounced gliosis than wild-type TDP-43 overexpression. Data are represented as mean ± SD. *Scale bars* 50 μm. *P < 0.05; **P < 0.01; ***P < 0.001

Interestingly, these pathological changes were accompanied by a 1.4-fold increase in caspase-3 expression levels in both Mt-TAR6/6 and Wt-TAR4/4 mice compared to Ntg mice (Fig. S5; *P* = 0.017 and *P* = 0.008, respectively). These data were confirmed by increased cleaved caspase-3 immunoreactivity for both cortical (Fig. S5) and spinal cord (data not shown) neurons in end-stage mutant and wild-type TDP-43 mice. Consistent with the fact that neurodegeneration is regularly accompanied by strong astroglial and microglial pathology, we also identified dose-dependent astrogliosis and microgliosis in mutant TDP-43 mice in the same regions that were affected by neuronal loss (Fig. 2a, b and Fig. S4b, c). Compared to Ntg mice, a statistically significant two- to fivefold increase in glial fibrillary acidic protein (GFAP)-positive astrocytes and Iba-1-positive microglial cells was observed in the highest expressing Mt-TAR6/6 mice in both motor cortex (*P* ≤ 0.001 for both) and spinal cord (*P* = 0.003 for both) (Fig. 2c, d). Interestingly, compared to Wt-TAR4/4 mice, Mt-TAR6/6 mice also showed a ≈50 % increased astroglial reactivity in the motor cortex (Fig. 2c; *P* = 0.034). Severe astrogliosis and microgliosis were also present in the hippocampus of end-stage Mt-TAR5/6 and Mt-TAR6/6 mice compared to control littermates, which is in accordance with the amount of neurodegeneration found in the different CA regions (Fig. S4b, c). These data suggest that the observed astrogliosis and microgliosis in TDP-43 mice are both dose- and mutation-dependent (Fig. 2c, d).

### Accumulation of Ubiquitinated Proteins and Eosinophilic Mitochondrial Aggregates in Mutant and Wild-Type TDP-43 Mice

TDP-43 is processed and degraded by both autophagy and the ubiquitin–proteasome system (UPS) and also acts as a maintenance factor of the autophagy system [37, 38]. Interestingly, increased lysosomal cysteine protease cathepsin D expression levels (1.5- and twofold increase) and immunostaining were observed in both brain and spinal cord of Mt-TAR6/6 and Wt-TAR4/4 mice compared to age-matched controls (Fig. S6a). However, utilizing both IHC and immunoblot analysis of total brain lysates, no significant alterations in either LC3 (microtubule-associated protein light chain 3) or p62 (autophagy receptor protein) were observed for Mt-TAR6/6 and Wt-TAR4/4 mice. These data suggest that overexpression of TDP-43 does not lead to an impaired turnover of autophagosomal vesicles.

**Fig. 3:**
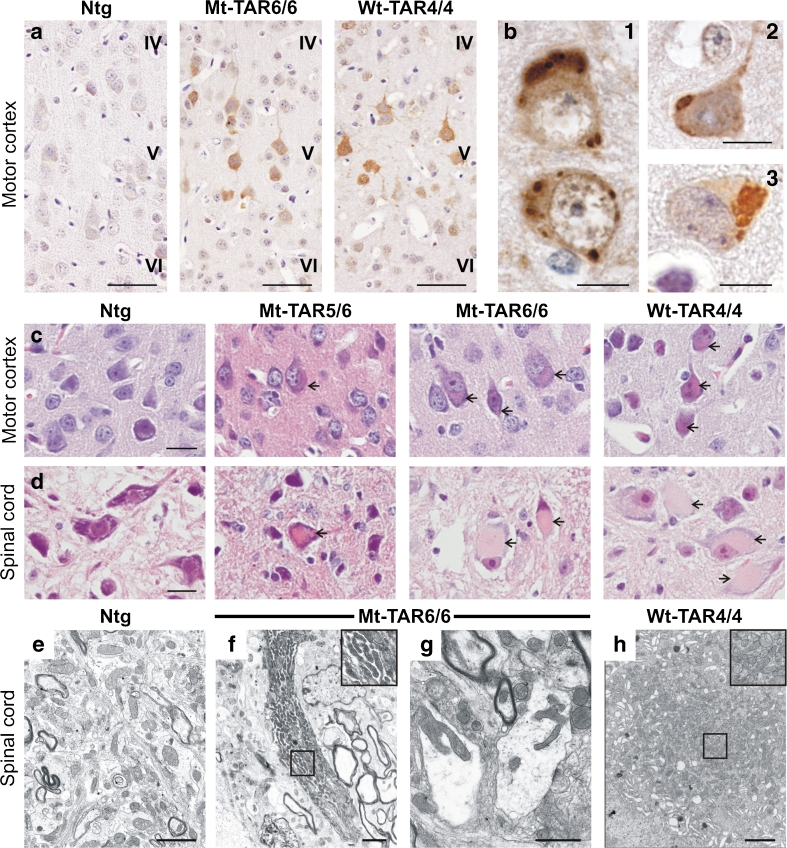
Accumulation of ubiquitinated proteins and eosinophilic mitochondrial aggregates in cortical and spinal neurons of transgenic TDP-43 mice. **a** Ubiquitin pathology in layer V of the motor cortex of both Mt-TAR6/6 and Wt-TAR4/4 mice, which was absent in Ntg mice. *Scale bars* 50 μm. **b** In addition, ubiquitinated cytoplasmic inclusions could be observed in (*1*) Mt-TAR5/6, (*2*) Mt-TAR6/6 and (*3*) Wt-TAR4/4 mice. *Scale bars* 10 μm. Hematoxylin and eosin (H&E) staining of brain and spinal cord showed accumulation of eosinophilic structures (*arrows*) in large motor neurons of **c** cortical layer V and **d** spinal cord of end-stage Mt-TAR6/6, Mt-TAR5/6, and Wt-TAR4/4 mice, which were absent in Ntg control mice. *Scale bars* 20 μm. **e**–**h** Ultrastructural analysis of lumbar spinal cord demonstrated abnormal accumulations of mitochondria of various shapes (*insets*) in **f**–**g** Mt-TAR6/6 and **h** Wt-TAR4/4 mice compared to **e** Ntg controls. **g** Clustered mitochondria were deformed and enlarged with disorganized cristae in Mt-TAR6/6 mice. **f** and **h** *Insets in the upper right corner* show a higher magnification of the boxed region. *Scale bars* 2 μm

Ubiquitinated neuronal inclusions are a pathological hallmark of several neurodegenerative disorders including ALS and FTLD-TDP [39]. To determine whether overexpression of mutant TDP-43 recapitulates the formation of these accumulations in our mutant TDP-43 mice, we examined brain and spinal cord sections of Mt-TAR6/6 and Mt-TAR5/6 mice and compared them to the pathology found in Wt-TAR4/4 mice as well as to ALS-FTLD patients. Ubiquitin IHC revealed an abnormal increase in cytoplasmic ubiquitin staining within motor neurons of the cortical layer V of Mt-TAR6/6 and Wt-TAR4/4 mice, which was absent in Ntg mice (Fig. 3a). Mildly increased ubiquitin immunoreactivity was also observed in other brain regions such as the hippocampus, pons, and Purkinje cells of the cerebellum. The intensity of the cytoplasmic ubiquitin immunoreactivity observed in Mt-TAR5/6 mice was highly variable, whereby mice having severe phenotype at younger ages developed stronger ubiquitin immunoreactivity compared to select mice that had milder phenotype at older ages. These data suggest that the diffuse neuronal ubiquitin immunoreactivity in TDP-43 mice is intimately associated with the disease progression.

In addition to the diffuse neuronal ubiquitin immunoreactivity, circumscribed ubiquitin-positive inclusions were also observed in cortical layer V neurons of several Mt-TAR5/6, Mt-TAR6/6, and WT-TAR4/4 mice but were absent in Ntg controls (Fig. 3b1–3). On several occasions, more than one inclusion was found in a single neuron. Spinal motor neurons also showed a diffuse ubiquitin staining in the cytoplasm together with an accumulation of ubiquitinated proteins for all analyzed mouse lines. However, in contrast to the brain, no clear ubiquitinated inclusions were observed in spinal cord sections. In addition, not all analyzed end-stage Mt-TAR6/6 mice developed ubiquitinated inclusions in contrast to the diffuse ubiquitin immunoreactivity that was always present in mice that had a severe phenotype (see above). Furthermore, few ≈ 1-month-old Mt-TAR5/6 mice showed more abundant and larger ubiquitin-positive accumulations in the motor cortex compared to younger and higher transgene-expressing Mt-TAR6/6 mice (Fig. 3b1–2). Of all TDP-43 mice, end-stage Wt-TAR4/4 mice showed the largest load of ubiquitinated inclusions in the motor cortex, and interestingly, Wt-TAR4/4 mice lived longer than the Mt-TAR6/6 mice (Fig. 3b 3). Taken together, these results suggest that development of inclusions is not essential for TDP-43-led pathology in TDP-43 transgenic mice.

Mt-TAR6/6 as well as Wt-TAR4/4 mice also presented with different inclusions that were ubiquitin-negative. These large, amorphous, eosinophilic inclusions were observed in the cytoplasm of the anterior horn cells of the spinal cord and coincided with development of severe pathology (Fig. 3d, arrows). In addition, the eosinophilic inclusions were also observed, although to a lesser extent, in cortical layer V (Fig. 3c, arrows), thalamus, pons, and the Purkinje cell layer of the cerebellum. No differences were identified for the eosinophilic aggregate load or size in the highest expressing Mt-TAR6/6 mice compared to equal expressing Wt-TAR4/4 mice (Fig. 3c, d). Further analysis of the nature of these cytoplasmic inclusions by electron microscopy for the same spinal cord regions showed that they coincided with abnormal accumulations of proliferating mitochondria (Fig. 3e–h), as was also recently shown in other TDP-43 models [19, 25, 28]. The majority of mitochondria in both mutant and wild-type TDP-43 mice had an abnormal ultrastructural appearance, comprising deformed cristae and fission deficits. Interestingly, despite the high load of abnormal mitochondria present in Mt-TAR6/6 mice, they never exhibited the same load of mitochondria as observed in Wt-TAR4/4 mice (Fig. 3e–h).

Consistent with the abnormal accumulation of ubiquitinated proteins in both mutant and wild-type TDP-43 mice, we also observed increased cytoplasmic reactivity to ubiquilin 2 (UBQLN2) that regulates degradation of ubiquitinated proteins. Interestingly, mutations in *UBQLN2* were found to cause chromosome X-linked ALS and ALS/dementia [40]. Although no UBQLN2-positive aggregates were observed in TDP-43 mice, UBQLN2 accumulation was detected in both cortical layer V and spinal motor neurons of Mt-TAR6/6 mice (Fig. S6b), and at least for cortical layer V, the staining patterns were appreciably stronger compared to Wt-TAR4/4 mice (Fig. S6b). Our results suggest that degradation of ubiquitinated proteins is disturbed in mutant and wild-type TDP-43 mice and more so in the mutant mice.

### TDP-43 Pathological Alterations in Mutant TDP-43 Overexpression Mice

**Fig. 4:**
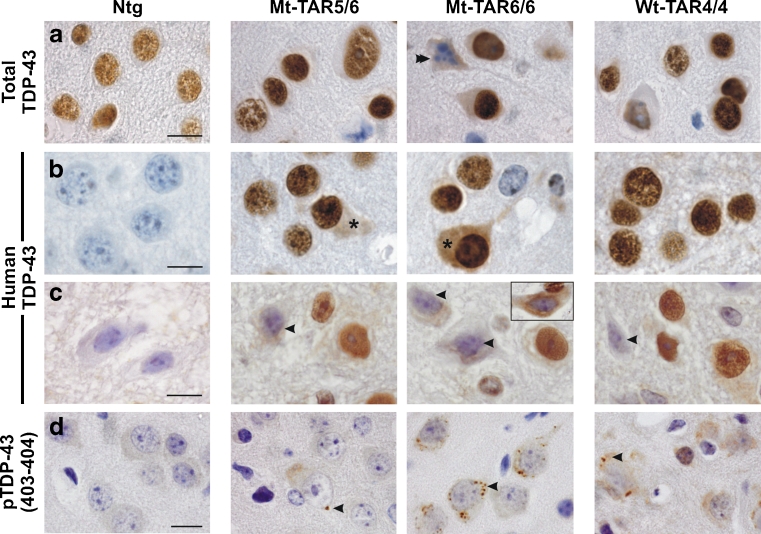
Redistribution of nuclear TDP-43 and formation of phosphorylated (pS403/404) TDP-43 accumulations in the cortex of mutant TDP-43 mice. Immunohistochemistry for **a** total and **b**–**c** human TDP-43 of Mt-TAR6/6, Mt-TAR5/6, and Wt-TAR4/4 mice indicated that TDP-43 resides mainly in the nucleus of **a**–**b** cortical and **c** spinal motor neurons. However, nuclear TDP-43 was absent in **a** apoptotic neurons (*double arrowhead*). **b** Using a human-specific TDP-43 antibody, some neurons of Mt-TAR6/6 and Mt-TAR5/6 mice also showed diffuse reactivity in the cytoplasm (*asterisks*). Human TDP-43 reactivity was absent in Ntg control mice. **c** The presence of cytoplasmic TDP-43 staining was often accompanied by reduced nuclear TDP-43 immunoreactivity in spinal motor neurons (*arrowheads*). Nuclear clearing and diffuse cytoplasmic staining were also observed using a non-species-specific antibody (*inset*). **d** Small granular structures immunoreactive for phosphorylated (pS403/404) TDP-43 were present within the cytoplasm of cortical neurons of Mt-TAR6/6 and Wt-TAR4/4 mice and, to a lesser extent, in Mt-TAR5/6 mice (*arrowheads*). *Scale bars* 10 μm

TDP-43 is suggested to be a principal component of ubiquitin-positive inclusions in ALS and FTLD-TDP [1, 2]. Using different TDP-43 antibodies (Table S1), the primary localization of TDP-43 was observed within neuronal nuclei of the brain and spinal cord of mutant and wild-type TDP-43 mice, as was the case for endogenous TDP-43 in Ntg mice (Fig. 4a, b). Nuclear TDP-43 was absent from apoptotic and dying neurons (Fig. 4a, double arrowhead) [12, 41]. Interestingly, a small subset of cortical neurons of Mt-TAR6/6 and Mt-TAR5/6 mice also showed diffuse cytoplasmic TDP-43 staining, which was more evident with a human-specific TDP-43 antibody (Fig. 4b, asterisks). Such diffuse cytoplasmic TDP-43 staining was also present in spinal motor neurons of Mt-TAR6/6 and Mt-TAR5/6 mice and many neurons also showed a strong reduction in nuclear TDP-43 immunoreactivity (Fig. 4c, arrowheads). Remarkable nuclear TDP-43 clearing was observed in the spinal cord of Mt-TAR6/6 mice using a TDP-43 non-species-specific antibody (Fig. 4c, inset). Utilizing a phosphorylated TDP-43 (pS403/pS404) specific antibody, small granular accumulations were also identified in the cytoplasm of cortical layer V neurons of both mutant and wild-type TDP-43 mice but were most abundant in the highest expressing Mt-TAR6/6 mice (Fig. 4d, arrowheads). Such accumulations were extremely rare in spinal motor neurons and absent in both cortical and spinal neurons of Ntg controls. More interestingly, neither TDP-43 nor phosphorylated TDP-43 immunostaining substantially colocalized with ubiquitin inclusions for mutant TDP-43 mice as has also been shown earlier for wild-type TDP-43 mice [24] (Fig. S7a–b). These findings suggest that an entirely different subset of proteins is ubiquitinated in TDP-43-mediated neurodegeneration in TDP-43 transgenic mice.

Because aggregation of FUS and TDP-43 has been suggested to proceed through the stress granule pathway [42–44], we analyzed any recruitment of stress granules in cytoplasmic phosphorylated TDP-43 granules. Utilizing a well-known marker for stress granules (T cell intracellular antigen-1, TIA-1), no clear colocalization was observed with the pS403/404-positive granules in the cortex of Mt-TAR6/6 mice (Fig. S7c). This data is in line with previous results, where in contrast to spinal cord regions, no colocalization of stress granule markers was present in TDP-43 inclusions in brains of FTLD-TDP and ALS-TDP patients [45]. As Mt-TAR6/6 mice do not show phosphorylated TDP-43 granules in the spinal cord, recruitment in stress granules could not be analyzed here.

**Fig. 5:**
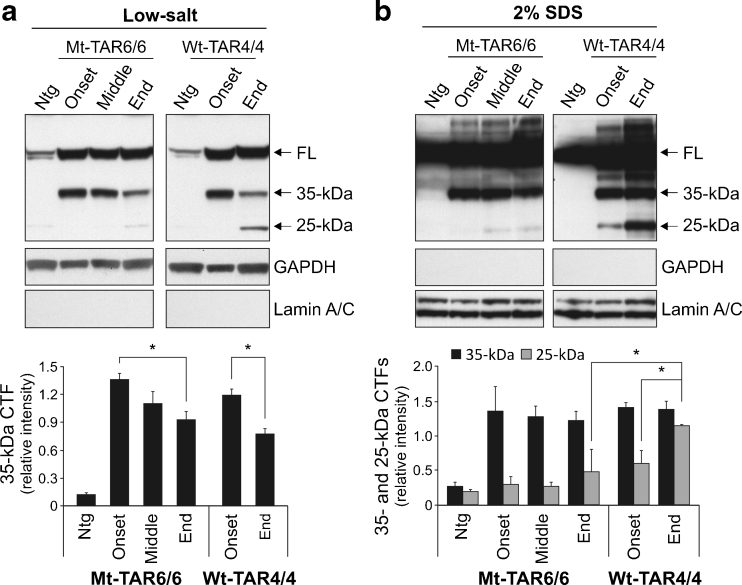
Aberrant processing of TDP-43 in mutant TDP-43 mice. **a**–**b** Immunoblot analysis of brain lysates of Mt-TAR6/6 and Wt-TAR4/4 mice using a non-species-specific TDP-43 antibody showed gradually decreased 35-kDa C-terminal fragments (CTFs) with disease progression in **a** low-salt fractions. **b** No major differences in ≈ 35-kDa CTFs were observed between disease onset and disease end stage in 2 % SDS fractions. Accumulation of ≈ 25-kDa CTFs in 2 % SDS fractions was more pronounced in Wt-TAR4/4 mice compared to Mt-TAR6/6 mice. **a**–**b** Quantification indicated no significant difference in Mt-TAR6/6 mice for ≈ 25-kDa CTFs between disease onset and disease end-stage. GAPDH and lamin A/C were used as a marker for low-salt and 2 % SDS fractions, respectively. Data are represented as mean ± SD. *P < 0.05

Pathological TDP-43 is also known to be cleaved into 25–35-kDa CTFs in the cortex, but not in the spinal cord of FTLD and ALS patients [1, 46, 47]. To similarly characterize the pathogenic potential of TDP-43 CTFs, we performed immunoblotting on sequential brain extracts from different disease stages of Mt-TAR6/6 mice and Wt-TAR4/4 mice. Utilizing a non-species-specific TDP-43 antibody, we identified ≈ 35-kDa fragments in soluble low-salt fractions already at disease onset, which decreased gradually with disease progression (Fig. 5a). Compared to disease onset, end-stage Mt-TAR6/6 mice showed a significant decrease in ≈ 35-kDa CTFs up to 32 % (Fig. 5a; *P* = 0.048) and was highly comparable with a similar statistically significant 35 % decrease in Wt-TAR4/4 mice in the low-salt fractions (Fig. 5a; *P* = 0.034). The low-salt fraction generally represents the cytoplasmic fraction with presence of cytoplasmic GAPDH and absence of nuclear lamin A/C proteins in this fraction [24]. Analysis of the sequentially extracted brain proteins in 2 % SDS fractions did not show notable differences in ≈ 35-kDa CTFs between disease onset and disease end-stage for both Mt-TAR6/6 and Wt-TAR4/4 mice (Fig. 5b). These data confirm earlier reports that the ≈ 35-kDa TDP-43 CTFs might not be critical for TDP-43 proteinopathy [1, 24].

On the other hand, accumulation of 25-kDa CTFs associated with disease severity was very remarkable for Wt-TAR4/4 mice especially in the insoluble 2 % SDS fraction (48 % increase; *P* = 0.023) and confirms our earlier report [24]. Interestingly, no significant difference in 25-kDa CTFs was observed for disease progression in Mt-TAR6/6 mice, and end-stage Mt-TAR6/6 mice showed 58 % lower levels of 25-kDa CTFs, compared to end-stage Wt-TAR4/4 mice (*P* = 0.011). Although we cannot exclude the possibility that toxicity incurred by mutant 25-kDa CTFs might be substantially higher than by wild-type fragments, our data already suggest that pathogenic mutations in TDP-43 do not induce an appreciably increased accumulation of CTFs by increased production or stability of these fragments in mice.

## Discussion

TDP-43 plays a major role in the pathogenesis of ALS and FTLD disorders characterized by TDP-43-positive neuropathology. While wild-type TDP-43 is the key pathogenic substrate in the majority of these ALS and FTLD patients, mutant forms of TDP-43 can also drive ALS pathology in those carrying a *TARDBP* missense mutation. To study the mechanism(s) by which TDP-43 causes ALS and FTLD, several mouse models expressing wild-type or mutant TDP-43 have been generated [18–28]. Interestingly, wild-type and mutant TDP-43 overexpressing mouse models show striking similarities in disease phenotype including gait impairment, neurodegeneration, and TDP-43 misprocessing, making it difficult to assess the relative contribution of mutated *TARDBP* to disease development. To address these questions, we established p.M337V human TDP-43 overexpression mouse models that expressed comparable TDP-43 levels to wild-type human TDP-43 mice, driven by the same Thy-1.2 promoter [24]. Thy-1.2 is active only after P8 and therefore avoids drastic effects of TDP-43 expression during development or very early postnatal stages [26, 48, 49]. Phenotypic, biochemical, and pathological comparisons between mutant TDP-43 mouse lines and similar transgenic wild-type TDP-43 lines, in the same experimental setting, provided us with interesting conclusions regarding differences and commonalities between mutant and wild-type TDP-43-led pathology. First, compared to wild-type TDP-43, mutant TDP-43 mice showed worsened disease aspects including motor dysfunction and neurodegeneration accompanied by gliosis. Although we have only studied the p.M337V mutation, we presume that an increased toxic potential could be extrapolated to other TDP-43 mutations affecting the terminal glycine-region of TDP-43.

Secondly, we show here that TDP-43-positive inclusions, regularly observed in brain and spinal cord of ALS and FTLD patients [50], are not a major disease feature in the studied TDP-43 mouse models. This is in line with other reported TDP-43 models where TDP-43 inclusions were also mostly absent (Table 2) [18, 20–22, 24, 25, 28]. Utilizing phosphorylated TDP-43 antibodies, we did observe very few cytoplasmic phosphorylated TDP-43 granules. Although formation of phosphorylated TDP-43 granules did not correlate with disease progression in TDP-43 mice, these granules were more abundant in mutant TDP-43 compared to wild-type TDP-43 mice. On the other hand, diffuse neuronal ubiquitin immunoreactivity was the only neuronal marker that associated with disease progression in mutant TDP-43 mice. The more characteristic ubiquitin-positive inclusions were less pronounced in mutant TDP-43 mice compared to equally expressing wild-type TDP-43 mice and did not seem to be influenced by either the progression or severity of the disease or disease-related mortality. These data all suggest that formation of TDP-43- or ubiquitin-positive inclusions is rather a late event in disease pathogenesis. We cannot exclude, however, the possibility that the occurrence of TDP-43 inclusions can hasten disease pathogenesis and subsequently shorten disease duration as has been observed in sporadic ALS patients [51].

**Table 2:** Overview of transgenic TDP-43 overexpression mice

	Transgene	Promoter	Expression (upon mTDP-43)	Ubiquitin inclusions	TDP-43 inclusions	C-terminal fragments	Ref.
Wegorzewska et al. (2009)	p.A315T	PrP	3-fold	Present (brain and spinal cord)	Absent	25-kDa and 35-kDa CTFs (progression with disease)	[22]
Wils et al. (2010)	Wild-type	Thy-1.2	2-fold	Present (brain and spinal cord)	Rare (brain and spinal cord)	25-kDa and 35-kDa CTFs (progression with disease)	[24]
Tsai et al. (2010)	Wild-type (mTDP-43)	CaMKII	2-fold	15–20 % of neurons (brain)	15–20 % of neurons (brain)	25-kDa and 35-kDa CTFs	[23]
Shan et al. (2010)	Wild-type	Thy-1	3.6-fold (males)	Rare (brain and spinal cord)	Present	n.d.	[19]
			1.3-fold (females)				
Xu et al. (2010)	Wild-type	PrP	2.5-fold	Absent (increased reactivity)	Rare (brain and spinal cord)	25-kDa and 35-kDa CTFs	[25]
Xu et al. (2011)	p.M337V	PrP	2.7-fold	Absent (increased reactivity)	Rare (brain and spinal cord)	25-kDa and 35-kDa CTFs	[28]
Stallings et al. (2010)	Wild-type	PrP	Up to 8-fold	Present (brain and spinal cord)	Rare (brain and spinal cord)	25-kDa and 35-kDa CTFs	[20]
	p.M337V						
	p.A315T						
Igaz et al. (2011)	Wild-type	CaMKIIα	0.4–1.7-fold	Rare (brain)	Rare (<1 %)	Absent	[18]
Swarup et al. (2011)	Wild-type	TDP-43	3-fold	Present (mutants)	Present (mutants)	25-kDa and 35-kDa CTFs (mutants)	[27]
	p.A315T						
	p.G348C						
Tian et al. (2011)	Wild-type	CAG	n.d. (22 cDNA copies)	Absent	Absent	n.d.	[21]
	p.M337V						
Cannon et al. (2012)	Wild-type	CaMKIIα	Up to 3-fold	Present	Present	25-kDa and 35-kDa CTFs	[26]
Janssens et al. (present paper)	p.M337V	Thy-1.2	Up to 1.8-fold	Present (only in brain)	Phospho TDP-43 granules (brain)	25-kDa and 35-kDa CTFs (progression with disease)	/
	(Cfr. wild-type; Wils et al., 2010)						

*n*.*d*. not determined

Another characteristic of TDP-43 proteinopathy is the accumulation of TDP-43 CTFs in specific brain regions of FTLD and ALS patients, but not in spinal cord regions [46, 47]. Formation of CTFs in the brain was reported in several TDP-43 overexpression models and was found to correlate with disease progression [22, 24]. We showed here that while both mutant and wild-type TDP-43 mice accumulated 25-kDa CTFs, their formation was significantly less pronounced in mutant TDP-43 mice despite having a more severe phenotype. Although formation of CTFs might have an impact on disease pathogenesis [46, 52], our data suggest that TDP-43 fragmentation is not a prerequisite for disease development at least not in the studied TDP-43 mice. Whether the lack of TDP-43-positive inclusions in our mutant TDP-43 mice is due to the lower accumulation of TDP-43 CTFs or due to the suggested requirement of a “second-hit” remains unknown [53].

To conclude, we show here that compared to wild-type TDP-43, mutant human TDP-43 expression in mice leads to a worsened dose-dependent disease phenotype with respect to motor dysfunctions, neurodegeneration, phosphorylated TDP-43 pathology, and lethality. Our data also suggest that TDP-43 CTFs or TDP-43 or ubiquitin-positive aggregates are not a prerequisite for disease development in TDP-43 overexpressing mice.

## Electronic supplementary material

Below is the link to the electronic supplementary material.

ESM 1 (PDF 671 kb)

ESM 2 (AVI 6923 kb)

ESM 3 (AVI 8893 kb)

## References

[1] Neumann M, Sampathu DM, Kwong LK (2006). Ubiquitinated TDP-43 in frontotemporal lobar degeneration and amyotrophic lateral sclerosis. Science.

[2] Arai T, Hasegawa M, Akiyama H (2006). TDP-43 is a component of ubiquitin-positive tau-negative inclusions in frontotemporal lobar degeneration and amyotrophic lateral sclerosis. Biochem Biophys Res Commun.

[3] Geser F, Martinez-Lage M, Kwong LK, Lee VM, Trojanowski JQ (2009). Amyotrophic lateral sclerosis, frontotemporal dementia and beyond: the TDP-43 diseases. J Neurol.

[4] Mackenzie IR, Feldman HH (2005). Ubiquitin immunohistochemistry suggests classic motor neuron disease, motor neuron disease with dementia, and frontotemporal dementia of the motor neuron disease type represent a clinicopathologic spectrum. J Neuropathol Exp Neurol.

[5] Sreedharan J, Blair IP, Tripathi VB (2008). TDP-43 mutations in familial and sporadic amyotrophic lateral sclerosis. Science.

[6] Rutherford NJ, Zhang YJ, Baker M (2008). Novel mutations in TARDBP (TDP-43) in patients with familial amyotrophic lateral sclerosis. PLoS Genet.

[7] Janssens J, Kleinberger G, Wils H, Van Broeckhoven C (2011). The role of mutant TAR DNA-binding protein 43 in amyotrophic lateral sclerosis and frontotemporal lobar degeneration. Biochem Soc Trans.

[8] Cruts M, Theuns J, Van Broeckhoven C (2012). Locus-specific mutation databases for neurodegenerative brain diseases. Hum Mutat.

[9] Buratti E, Brindisi A, Giombi M, Tisminetzky S, Ayala YM, Baralle FE (2005). TDP-43 binds heterogeneous nuclear ribonucleoprotein A/B through its C-terminal tail: an important region for the inhibition of cystic fibrosis transmembrane conductance regulator exon 9 splicing. J Biol Chem.

[10] Buratti E, Baralle FE (2010). The multiple roles of TDP-43 in pre-mRNA processing and gene expression regulation. RNA Biol.

[11] Sephton CF, Cenik C, Kucukural A (2011). Identification of neuronal RNA targets of TDP-43-containing ribonucleoprotein complexes. J Biol Chem.

[12] Kumar-Singh S (2011). Progranulin and TDP-43: mechanistic links and future directions. J Mol Neurosci.

[13] Hasegawa M, Arai T, Nonaka T (2008). Phosphorylated TDP-43 in frontotemporal lobar degeneration and amyotrophic lateral sclerosis. Ann Neurol.

[14] Gitler AD, Shorter J (2011). RNA-binding proteins with prion-like domains in ALS and FTLD-U. Prion.

[15] Saini A, Chauhan VS (2011). Delineation of the core aggregation sequences of TDP-43 C-terminal fragment. ChemBioChem.

[16] Johnson BS, Snead D, Lee JJ, McCaffery JM, Shorter J, Gitler AD (2009). TDP-43 is intrinsically aggregation-prone, and amyotrophic lateral sclerosis-linked mutations accelerate aggregation and increase toxicity. J Biol Chem.

[17] Nonaka T, Kametani F, Arai T, Akiyama H, Hasegawa M (2009). Truncation and pathogenic mutations facilitate the formation of intracellular aggregates of TDP-43. Hum Mol Genet.

[18] Igaz LM, Kwong LK, Lee EB (2011). Dysregulation of the ALS-associated gene TDP-43 leads to neuronal death and degeneration in mice. J Clin Invest.

[19] Shan X, Chiang PM, Price DL, Wong PC (2010). Altered distributions of Gemini of coiled bodies and mitochondria in motor neurons of TDP-43 transgenic mice. Proc Natl Acad Sci USA.

[20] Stallings NR, Puttaparthi K, Luther CM, Burns DK, Elliott JL (2010). Progressive motor weakness in transgenic mice expressing human TDP-43. Neurobiol Dis.

[21] Tian T, Huang C, Tong J, Yang M, Zhou H, Xia XG (2011). TDP-43 potentiates alpha-synuclein toxicity to dopaminergic neurons in transgenic mice. Int J Biol Sci.

[22] Wegorzewska I, Bell S, Cairns NJ, Miller TM, Baloh RH (2009). TDP-43 mutant transgenic mice develop features of ALS and frontotemporal lobar degeneration. Proc Natl Acad Sci USA.

[23] Tsai KJ, Yang CH, Fang YH (2010). Elevated expression of TDP-43 in the forebrain of mice is sufficient to cause neurological and pathological phenotypes mimicking FTLD-U. J Exp Med.

[24] Wils H, Kleinberger G, Janssens J (2010). TDP-43 transgenic mice develop spastic paralysis and neuronal inclusions characteristic of ALS and frontotemporal lobar degeneration. Proc Natl Acad Sci USA.

[25] Xu YF, Gendron TF, Zhang YJ (2010). Wild-type human TDP-43 expression causes TDP-43 phosphorylation, mitochondrial aggregation, motor deficits, and early mortality in transgenic mice. J Neurosci.

[26] Cannon A, Yang B, Knight J (2012). Neuronal sensitivity to TDP-43 overexpression is dependent on timing of induction. Acta Neuropathol.

[27] Swarup V, Phaneuf D, Bareil C, Robertson J, Rouleau GA, Kriz J, Julien JP (2011). Pathological hallmarks of amyotrophic lateral sclerosis/frontotemporal lobar degeneration in transgenic mice produced with TDP-43 genomic fragments. Brain.

[28] Xu YF, Zhang YJ, Lin WL, Cao X, Stetler C, Dickson DW, Lewis J, Petrucelli L (2011). Expression of mutant TDP-43 induces neuronal dysfunction in transgenic mice. Mol Neurodegener.

[29] Van Broeck B, Vanhoutte G, Pirici D (2008). Intraneuronal amyloid beta and reduced brain volume in a novel APP T714I mouse model for Alzheimer’s disease. Neurobiol Aging.

[30] Kumar-Singh S, Theuns J, Van Broeck B (2006). Mean age-of-onset of familial Alzheimer disease caused by presenilin mutations correlates with both increased Abeta42 and decreased Abeta40. Hum Mutat.

[31] Kumar-Singh S, Pirici D, McGowan E, Serneels S, Ceuterick C, Hardy J, Duff K, Dickson D, Van Broeckhoven C (2005). Dense-core plaques in Tg2576 and PSAPP mouse models of Alzheimer’s disease are centered on vessel walls. Am J Pathol.

[32] Pirici D, Vandenberghe R, Rademakers R (2006). Characterization of ubiquitinated intraneuronal inclusions in a novel Belgian frontotemporal lobar degeneration family. J Neuropathol Exp Neurol.

[33] Kollias G, Spanopoulou E, Grosveld F, Ritter M, Beech J, Morris R (1987). Differential regulation of a Thy-1 gene in transgenic mice. Proc Natl Acad Sci USA.

[34] Duchen L, Strich S, Falconer D (1964). Clinical and pathological studies of an hereditary neuropathy in mice (dystonia musculorum). Brain.

[35] Nadeau JH (2003). Modifier genes and protective alleles in humans and mice. Curr Opin Genet Dev.

[36] Ayala YM, De CL, Avendano-Vazquez SE (2011). TDP-43 regulates its mRNA levels through a negative feedback loop. EMBO J.

[37] Wang X, Fan H, Ying Z, Li B, Wang H, Wang G (2010). Degradation of TDP-43 and its pathogenic form by autophagy and the ubiquitin–proteasome system. Neurosci Lett.

[38] Bose JK, Huang CC, Shen CK (2011). Regulation of autophagy by neuropathological protein TDP-43. J Biol Chem.

[39] Lehman NL (2009). The ubiquitin proteasome system in neuropathology. Acta Neuropathol.

[40] Deng HX, Chen W, Hong ST (2011). Mutations in UBQLN2 cause dominant X-linked juvenile and adult-onset ALS and ALS/dementia. Nature.

[41] Kleinberger G, Wils H, Ponsaerts P, Joris G, Timmermans JP, Van Broeckhoven C, Kumar-Singh S (2010). Increased caspase activation and decreased TDP-43 solubility in progranulin knockout cortical cultures. J Neurochem.

[42] Liu-Yesucevitz L, Bilgutay A, Zhang YJ (2010). Tar DNA binding protein-43 (TDP-43) associates with stress granules: analysis of cultured cells and pathological brain tissue. PLoS One.

[43] Dormann D, Rodde R, Edbauer D (2010). ALS-associated fused in sarcoma (FUS) mutations disrupt Transportin-mediated nuclear import. EMBO J.

[44] Wolozin B (2012). Regulated protein aggregation: stress granules and neurodegeneration. Mol Neurodegener.

[45] Bentmann E, Neumann M, Tahirovic S, Rodde R, Dormann D, Haass C (2012). Requirements for stress granule recruitment of fused in sarcoma (FUS) and TAR DNA-binding protein of 43 kDa (TDP-43). J Biol Chem.

[46] Igaz LM, Kwong LK, Chen-Plotkin A, Winton MJ, Unger TL, Xu Y, Neumann M, Trojanowski JQ, Lee VM (2009). Expression of TDP-43 C-terminal fragments in vitro recapitulates pathological features of TDP-43 proteinopathies. J Biol Chem.

[47] Igaz LM, Kwong LK, Xu Y (2008). Enrichment of C-terminal fragments in TAR DNA-binding protein-43 cytoplasmic inclusions in brain but not in spinal cord of frontotemporal lobar degeneration and amyotrophic lateral sclerosis. Am J Pathol.

[48] Larsen DD, Callaway EM (2006). Development of layer-specific axonal arborizations in mouse primary somatosensory cortex. J Comp Neurol.

[49] Li M, Cui Z, Niu Y, Liu B, Fan W, Yu D, Deng J (2010). Synaptogenesis in the developing mouse visual cortex. Brain Res Bull.

[50] Lee EB, Lee VM, Trojanowski JQ (2012). Gains or losses: molecular mechanisms of TDP43-mediated neurodegeneration. Nat Rev Neurosci.

[51] Nishihira Y, Tan CF, Toyoshima Y, Yonemochi Y, Kondo H, Nakajima T, Takahashi H (2009). Sporadic amyotrophic lateral sclerosis: widespread multisystem degeneration with TDP-43 pathology in a patient after long-term survival on a respirator. Neuropathology.

[52] Zhang YJ, Xu YF, Cook C (2009). Aberrant cleavage of TDP-43 enhances aggregation and cellular toxicity. Proc Natl Acad Sci USA.

[53] Pesiridis GS, Tripathy K, Tanik S, Trojanowski JQ, Lee VM (2011). A “two-hit” hypothesis for inclusion formation by carboxyl-terminal fragments of TDP-43 protein linked to RNA depletion and impaired microtubule-dependent transport. J Biol Chem.

