# Supplementary material for: Overexpression of ALS-Associated p.M337V Human TDP-43 in Mice Worsens Disease Features Compared to Wild-type Human TDP-43 Mice
Source: Mol Neurobiol. 2013 Mar 10;48(1):22–35. doi: 10.1007/s12035-013-8427-5 (PMC3718993; doi:10.1007/s12035-013-8427-5)
Supplement: Supplementary file 1 — (PDF 671 kb) [file 12035_2013_8427_MOESM1_ESM.pdf]

Overexpression of ALS associated p.M337V human TDP-43 in mice worsens disease features compared to wild-type human TDP-43 mice

Jonathan Janssens[a, b], Hans Wils[a, b], Gernot Kleinberger[a, b], Geert Joris[a, b], Ivy Cuijt[a, b], Chantal Ceuterick-de Groote [d], Christine Van Broeckhoven[a, b*], Samir Kumar-Singh[a,b*]

[a] Neurodegenerative Brain Diseases Group, Department of Molecular Genetics, VIB, Antwerp, Belgium; [b] Laboratory of Neurogenetics Institute Born-Bunge, University of Antwerp, Antwerp, Belgium; [c] University of Antwerp, Antwerp, Belgium; [d] Laboratory of Ultrastructural Neuropathology Institute Born-Bunge, University of Antwerp, Antwerp, Belgium

***Corresponding authors:**

Reprint and information requests to Prof. Dr. Christine Van Broeckhoven, PhD, DSc, Neurodegenerative Brain Diseases Group, Department of Molecular Genetics, VIB, , Universiteitsplein 1, B-2610, Antwerp, Belgium; Tel: +3232651102; Fax: +3232651112; Email: christine.vanbroeckhoven@molgen.vib-ua.be

Information requests to Prof. Dr. Samir Kumar-Singh, MD, PhD, **Current address**: Molecular and Cellular Neuropathology Group, Laboratory of Cell Biology and Histology, Translational Neuroscience Department, Faculty of Medicine, University of Antwerp - CGB, Groenenborgerlaan 171, B-2020 Antwerp, Belgium; Tel: +3232653329; Fax: +32-3032653301 Email: samir.kumarsingh@ua.ac.be

Supplementary Information

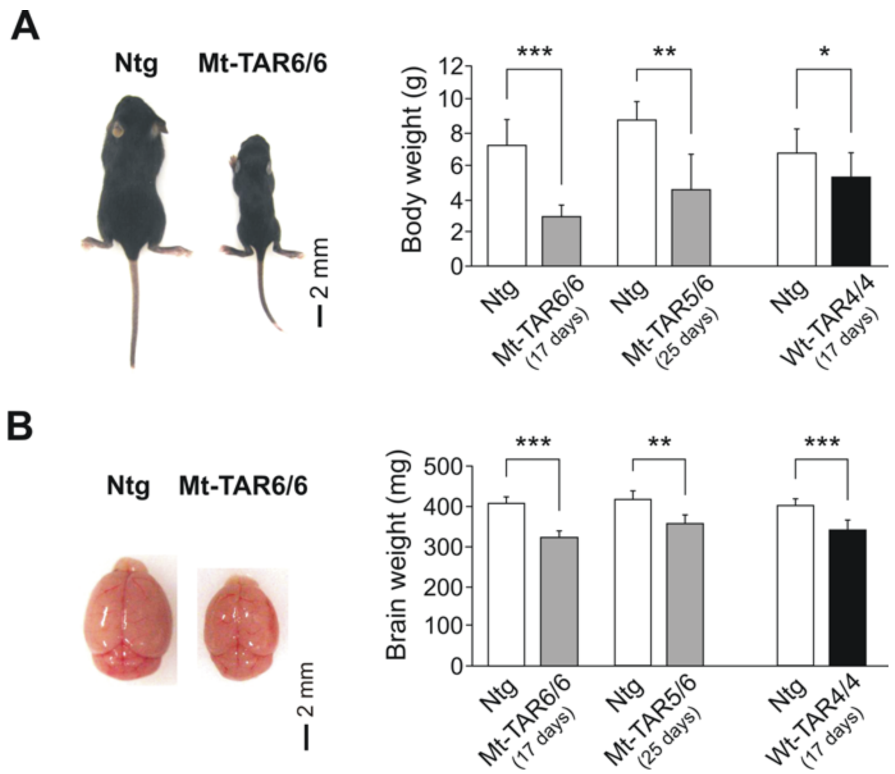

Fig. S1 Postnatal growth retardation in mutant and wild-type TDP-43 expressing mice. Early postnatal growth retardation in transgenic mice expressing either wild-type or mutant TDP-43. Both mouse models showed a significant reduction in (***A***) body weight of Mt-TAR6/6 (54%), Mt-TAR5/6 (48%) and Wt-TAR4/4 (21%) mice. (***B***) In addition, brain weight was significantly reduced in Mt-TAR6/6 (22%), Mt-TAR5/6 (15%) and Wt-TAR4/4 (15%) mice compared to Ntg littermates. Data are represented as mean ± SD. *P <0.05; **P < 0.01; ***P < 0.001.

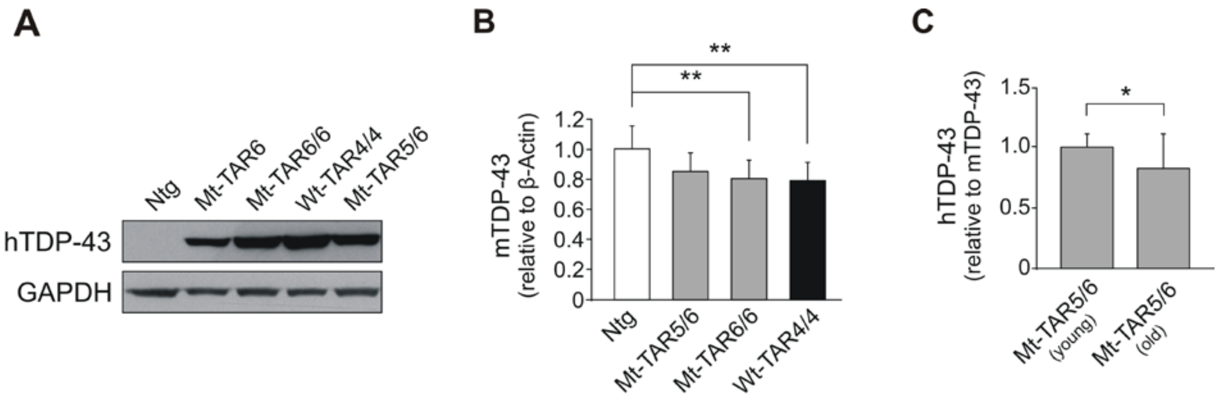

Fig. S2 Overexpression of mutant and wild-type TDP-43 in neurons leading to downregulation of endogenous TDP-43. (***A***) Brain immunoblotting with a human-specific TDP-43 antibody shows higher human TDP-43 (hTDP-43) protein levels in Wt-TAR4/4 mice compared with Mt-TAR6/6 mice, Mt-TAR5/6 and Ntg littermates. (***B***) mRNA expression levels in brain of endogenous TDP-43 (mTDP-43) measured by qRT-PCR (relative to β-Actin transcript levels) using primers specific to mTDP-43. Transgenic mice show a dose-dependent downregulation of mTDP-43 transcript levels, relative to Ntg littermates. Data analysis revealed a downregulation of 18% (Mt-TAR5/6), 24% (Mt-TAR6/6) and 26% (Wt-TAR4/4). (***C***) Significant difference in brain for hTDP-43 transcript levels (relative to mTDP-43 transcript levels) between Mt-TAR5/6 mice that develop an ALS-FTLD-like phenotype at young and at older ages, implying the involvement of genetic or epigenetic modifying factors that influence TDP-43 levels. Data are represented as mean ± SD. **P* <0.05; ***P* < 0.01.

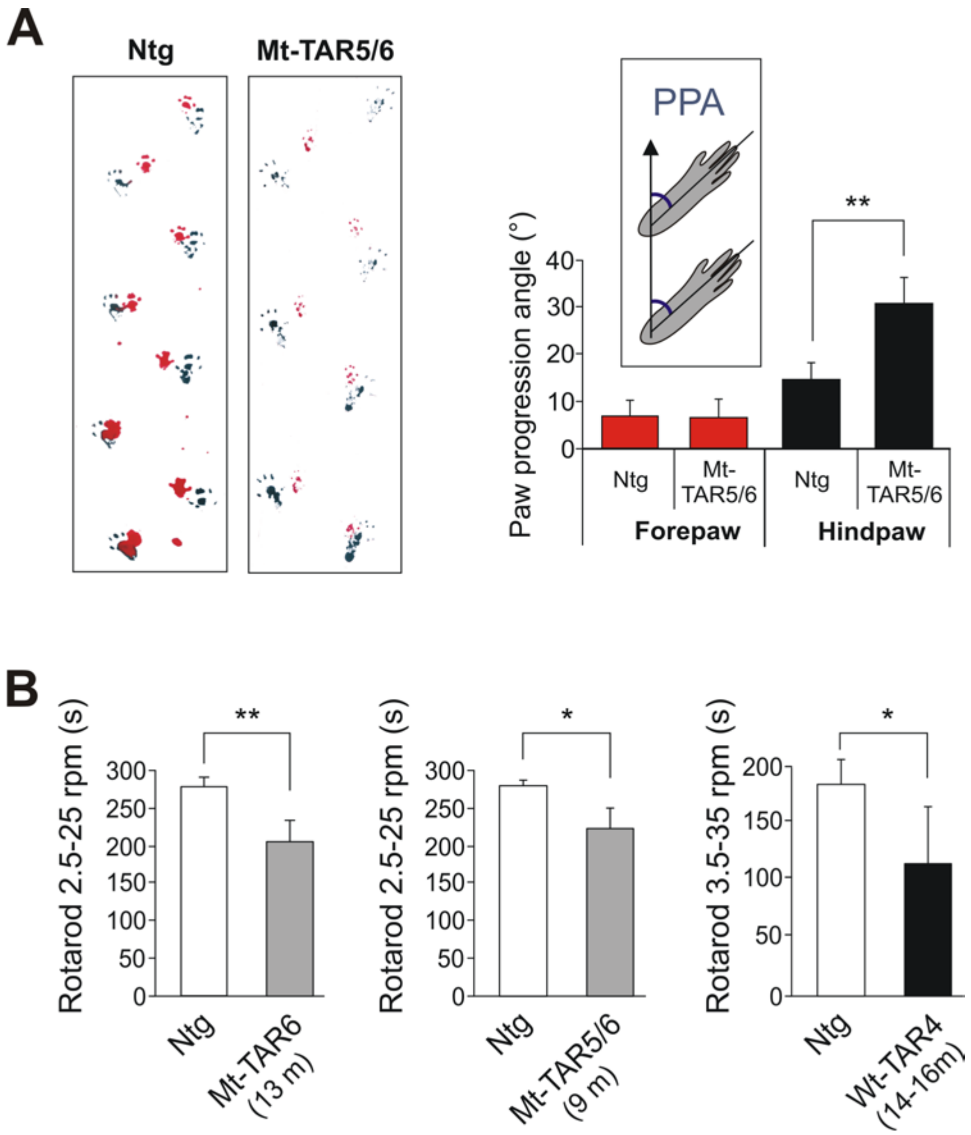

Fig. S3 Neuronal expression of mutant TDP-43 induced a reduced motor performance in mutant TDP-43 mice. (***A***) Gait patterns of Ntg and Mt-TAR5/6 mice were obtained by dipping mouse paws in red (forepaws) and black (hindpaws) ink. The paw progression angle (PPA), the angular difference between the axis of the paw and the line of progression (inset), was increased for the hindlimbs but not for the forelimbs in mutant mice compared to Ntg littermates. This measure is indicative for the development of paralysis in the hindlimbs. (***B***) Significant reduction in general motor performance measured on accelerated rotarod for Mt-TAR6, Mt-TAR5/6 and Wt-TAR4/4 mice compared to Ntg littermates. Data are represented as mean ± SD. $^*P<0.05$; $^{**}P<0.01$.

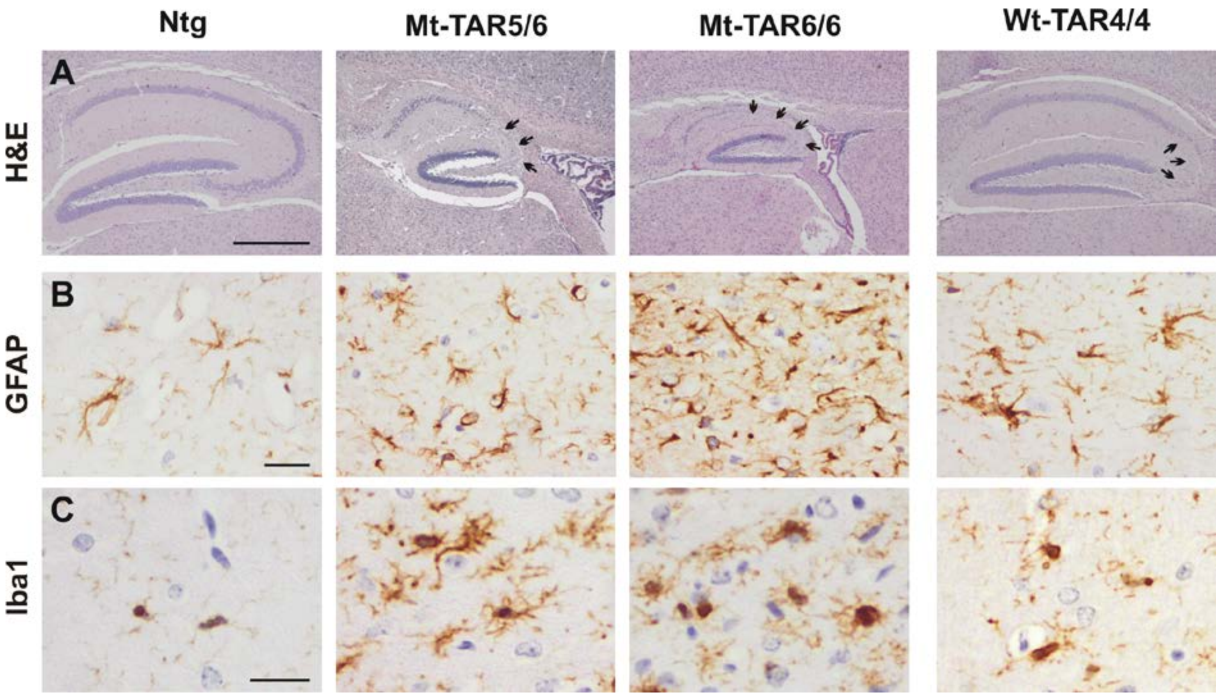

Fig. S4 Increased gliosis and neurodegeneration in the hippocampus of mutant TDP-43 mice. (***A***) Overexpression of mutant TDP-43 induced a more severe neurodegeneration in several CA fields of the hippocampus (arrows) compared to wild-type TDP-43 mice, which depended on TDP-43 dose. Scale bars 500µm. Highly increased (***B***) astrogliosis and (***C***) microgliosis in the hippocampus of Mt-TAR6/6 mice compared to Ntg littermates. Upregulation was more pronounced in mutant compared to wild-type TDP-43 mice. Scale bars 20µm.

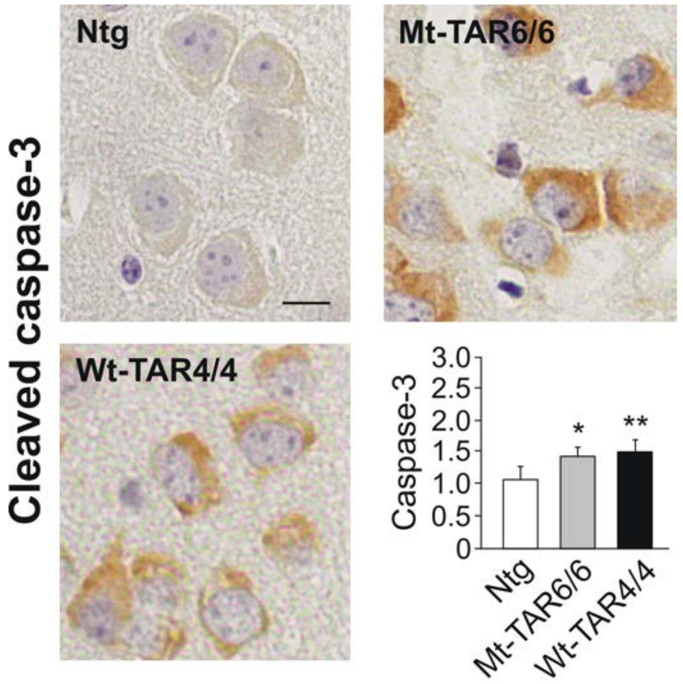

Fig. S5 Increased apoptosis in mutant and wild-type TDP-43 mice. Increased cleaved caspase-3 immunoreactivity was observed in cortical neurons of both Mt-TAR6/6 and Wt-TAR4/4 mice compared to Ntg mice. This is also the area that showed the most severe ubiquitin pathology. Caspase-3 transcript levels were significantly increased in brain of Mt-TAR6/6 and Wt-TAR4/4 mice compared to Ntg controls. Scale bars 10 µm. *$P < 0.05$; **$P < 0.01$.

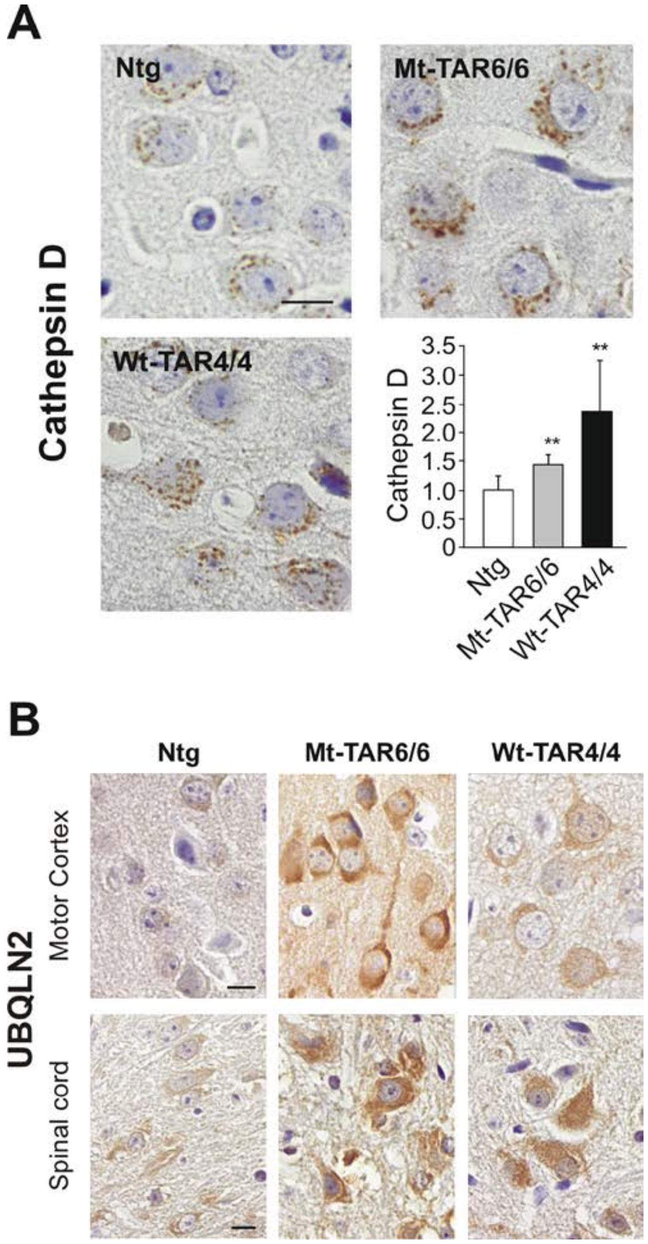

Fig. S6 Cathepsin D and Ubiquilin 2 pathology in neurons of mutant TDP-43 mice. (***A***) Immunohistochemistry for the lysosomal protease cathepsin D showing increased reactivity in the cortex of end-stage Mt-TAR6/6 and Wt-TAR4/4 mice. qRT-PCR analysis confirms significantly increased transcript levels of cathepsin D for Mt-TAR6/6 and Wt-TAR4/4 mice compared to Ntg controls. (***B***) Ubiquilin 2 immunoreactivity in brain and spinal cord was increased in mutant TDP-43 mice compared to Ntg control mice. Scale bars 10 µm. *$P <$ 0.05; **$P < 0.01$.

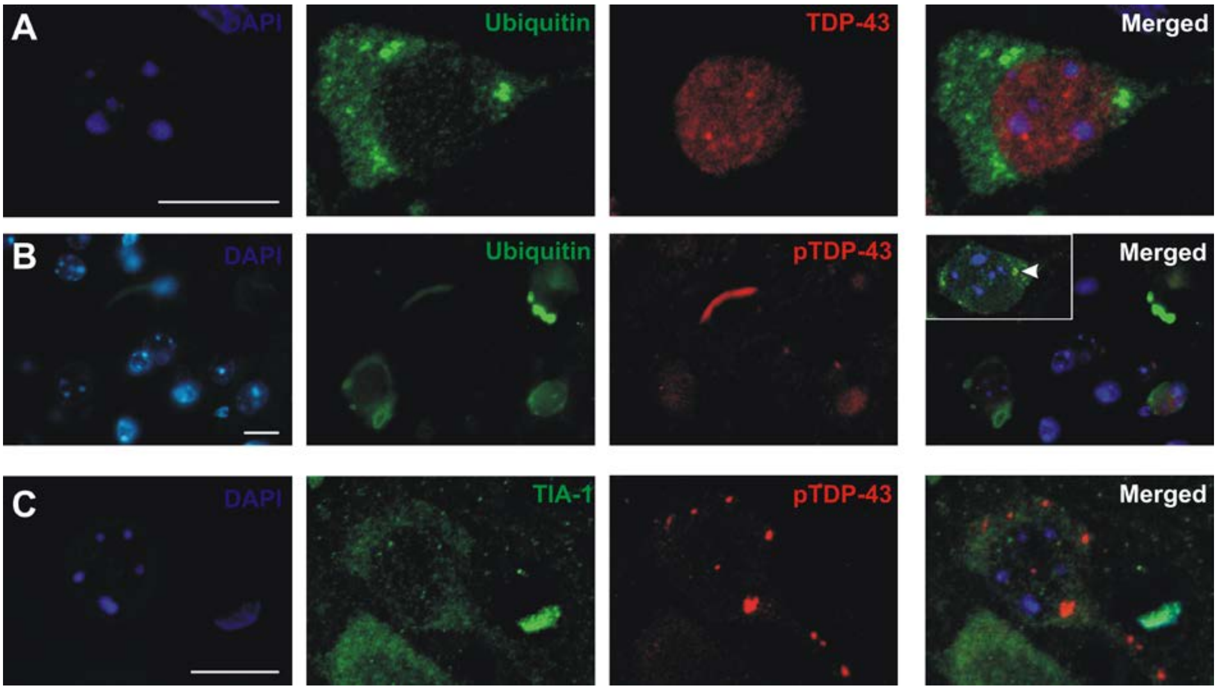

Fig. S7 Phosphorylated TDP-43 is not ubiquitinated and not recruited into stress granules in the cortex of mutant TDP-43 mice. (***A***-***B***) Double immunofluorescence of ubiquitin with a (***A***) non-species-specific TDP-43 and a (***B***) phospho-TDP-43-specific (pS409/410) antibody showing no co-localization in the cortex of mutant TDP-43 mice. However, very few punctate ubiquitin-positive inclusions showed co-localization with phosphorylated TDP-43 (inset, arrowhead). (***C***) Double immunofluorescence of a phospho-TDP-43-specific (pS403/404) antibody with T cell intracellular antigen 1 (TIA-1) as a marker for stress granules showing no co-localization in the cortex of mutant TDP-43 mice. Scale bars 10µm.

Table S1 Antibodies used for the characterization of TDP-43 overexpression mice

**Antibody**	**Epitope/Marker**	**Clonality**	**Species**	**Dilution**	**Application**	**Source**
anti-Cathepsin D (Clone N-19)	Lysosomal protease Cathepsin D	pAb	Goat	1/200	IHC	Santa Cruz Biotechnology
anti-Cleaved caspase-3	Caspase-3	pAb	Rabbit	1/1000	IHC	Cell Signaling Technology
anti-GAPDH	Glyceraldehyde 3-phosphate dehydrogenase	mAb	Mouse	1/200000	WB	Meridian Life Science
anti-GFAP	Glial fibrillary acidic protein	mAb	Mouse	1/20000	IHC	DAKO
anti-Iba1	Ionized calcium-binding adaptor molecule 1	pAb	Rabbit	1/2000	IHC	Wako Chemicals
anti-Lamin A/C (clone N-18)	Lamin A/C	mAb	Goat	1/1000	WB	Santa Cruz Biotechnology
anti-LC3b (D11 XP)	Microtubule-associated protein light chain 3	mAb	Rabbit	1/500	IHC	Cell Signaling Technology
anti-p62	p62 protein (Sequestosome-1)	pAb	Guinea pig	1/2000	IHC	Progen Biotechnik
anti-TDP-43	TDP-43	pAb	Rabbit	1/2000	WB, IHC	ProteinTech-Group
anti-Human TDP-43	TDP-43	mAb	Mouse	1/1000	IHC	Abnova
anti-PhosphoTDP-43 (pS403/404)	Phosphorylated TDP-43	pAb	Rabbit	1/500	IHC	Cosmo Bio
anti-PhosphoTDP-43 (pS409/410)	Phosphorylated TDP-43	pAb	Rabbit	1/500	WB, IHC	Cosmo Bio
anti-TIA-1 (C-20)	T cell intracellular antigen 1	pAb	Goat	1/100	IHC	Santa Cruz Biotechnology
anti-Ubiquitin	Ubiquitin	pAb	Rabbit	1/2000	IHC	DAKO
anti-Ubiquitin	Ubiquitin	mAb	Mouse	1/100	IHC	Life Technologies
anti-Ubiquilin 2	Ubiquilin 2	mAb	Mouse	1/1500	IHC	Novus Biologicals

Movie S1 Typical example of an end-stage paralysis in an 18-day-old Mt-TAR6/6 mice

Movie S2 About 5% of Mt-TAR6 mice developed a complete paralysis of the hindlimbs where animals were unable to hold their body off the ground and used their forelimbs to drag themselves forward.
